# N-Glycans on the Rift Valley Fever Virus Envelope Glycoproteins Gn and Gc Redundantly Support Viral Infection via DC-SIGN

**DOI:** 10.3390/v8050149

**Published:** 2016-05-23

**Authors:** Inaia Phoenix, Shoko Nishiyama, Nandadeva Lokugamage, Terence E. Hill, Matthew B. Huante, Olga A.L. Slack, Victor H. Carpio, Alexander N. Freiberg, Tetsuro Ikegami

**Affiliations:** 1 Department of Pathology, The University of Texas Medical Branch, Galveston, TX 77555, USA; inphoeni@utmb.edu (I.P.); shnishiy@utmb.edu (S.N.); nalokuga@utmb.edu (N.L.); tehill@utmb.edu (T.E.H.); olga.slack@novartis.com (O.A.L.S.); anfreibe@utmb.edu (A.N.F.); 2 Department of Microbiology and Immunology, The University of Texas Medical Branch, Galveston, TX 77555, USA; mbhuante@utmb.edu (M.B.H.); vhcarpio@utmb.edu (V.H.C.); 3 The Sealy Center for Vaccine Development, The University of Texas Medical Branch, Galveston, TX 77555, USA; 4 The Center for Biodefense and Emerging Infectious Diseases, The University of Texas Medical Branch, Galveston, TX 77555, USA; 5 Galveston National Laboratory, The University of Texas Medical Branch, Galveston, TX 77555, USA

**Keywords:** Rift Valley fever virus, *N-glycosylation*, Gn, Gc, sequon, DC-SIGN, L-SIGN

## Abstract

Rift Valley fever is a mosquito-transmitted, zoonotic disease that infects humans and ruminants. Dendritic cell specific intercellular adhesion molecule 3 (ICAM-3) grabbing non-integrin (DC-SIGN) acts as a receptor for members of the phlebovirus genus. The Rift Valley fever virus (RVFV) glycoproteins (Gn/Gc) encode five putative N-glycan sequons (asparagine (N)–any amino acid (X)–serine (S)/threonine (T)) at positions: N438 (Gn), and N794, N829, N1035, and N1077 (Gc). The *N*-glycosylation profile and significance in viral infection via DC-SIGN have not been elucidated. Gc *N*-glycosylation was first evaluated by using Gc asparagine (N) to glutamine (Q) mutants. Subsequently, we generated a series of recombinant RVFV MP-12 strain mutants, which encode N-to-Q mutations, and the infectivity of each mutant in Jurkat cells stably expressing DC-SIGN was evaluated. Results showed that Gc N794, N1035, and N1077 were *N*-glycosylated but N829 was not. Gc N1077 was heterogeneously *N*-glycosylated. RVFV Gc made two distinct *N*-glycoforms: “Gc-large” and “Gc-small”, and N1077 was responsible for “Gc-large” band. RVFV showed increased infection of cells expressing DC-SIGN compared to cells lacking DC-SIGN. Infection via DC-SIGN was increased in the presence of either Gn N438 or Gc N1077. Our study showed that *N*-glycans on the Gc and Gn surface glycoproteins redundantly support RVFV infection via DC-SIGN.

## 1. Introduction

Rift Valley fever (RVF) is a mosquito-borne, viral disease endemic to Africa and is characterized by high rates of abortion, fetal deformities, and high rates of newborn mortality, particularly in sheep, goats, and cattle [1]. Humans can be infected through close contact with the body fluids of infected animals or from the bites of infected mosquitoes [2]. Human RVF is typically characterized by a self-limiting febrile illness (e.g., biphasic fever, severe headaches, muscle pain, or nausea). However, some patients may develop more severe disease, such as lethal hemorrhagic fever, neurologic disorders, or blindness [3]. RVF is caused by Rift Valley fever virus (RVFV), which belongs to the genus *Phlebovirus* of the family *Bunyaviridae*. Because of the major social and economic impacts of the disease in both public health and agriculture, RVFV is classified as a Category A Priority Pathogen by National Institutes of Health (NIH)/National Institute of Allergy and Infectious Diseases (NIAID) and also listed as an overlap select agent by the United States Department of Health and Human Services (HHS) and the United States Department of Agriculture (USDA), which could pose a severe threat to public health and agriculture [4]. Currently, there is no approved, effective treatment for patients with RVFV infection, and the development of an antiviral treatment is of great importance to improve the prognosis of patients.

RVFV is a three-segmented, negative-stranded RNA virus, and the genome consists of the Large (L), Medium (M), and Small (S) segments [5]. The L-segment encodes the L protein, which is a RNA-dependent RNA polymerase. The S-segment encodes nucleoprotein (N) and a nonstructural protein (NSs), in an ambi-sense manner. The M-segment encodes the 78-kD protein, NSm, Gn (537 amino acids (aa), and Gc (507 aa) [6]. The 78-kD and NSm are accessory proteins and dispensable for viral replication [7,8]. The NSm protein is a minor virulence factor that delays apoptosis in infected cells [8,9,10]. The 78-kD protein plays a role in viral dissemination in mosquito vectors [11], and is incorporated into virions from infected mosquito C6/36 cells but not from Vero cells [12].

**Figure 1:**
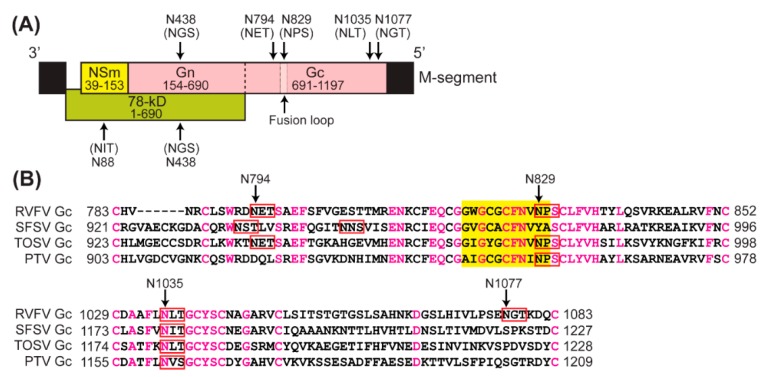
The asparagine (N)-any amino acid (X)-serine (S)/threonine (T) sequons of Rift Valley fever virus (RVFV) and other related phleboviruses. (**A**) Schematic diagram of the M-segment RNA and protein expressions. The RVFV medium (M)-segment encodes a single M mRNA, and co-translational cleavage and leaky ribosomal scanning of the initiation codons produce at least four proteins: 78-kD, nonstructural protein m (NSm) and glycoproteins Gn, and Gc. The Gn, and Gc are structural proteins, and the 78-kD protein is structural when virions are made in mosquito cells. There are six N-X-S/T sequons, which are potentially utilized for *N*-glycosylation: N88 (78-kD), N438 (Gn and 78-kD), N794 (Gc), N829 (Gc), N1035 (Gc), and N1077 (Gc); (**B**) Partial alignment of Gc amino acid sequences among RVFV, Sandfly fever Sicilian virus (SFSV: Genbank Accession No. U30500), Toscana virus (TOSV: Genbank Accession No. X89628), and Punta Toro virus (PTV: Genbank Accession No. DQ363407). Conserved amino acids are shown in pink, while the N-X-S/T sequons are shown in red squares. Positions of amino acids (aa.) at the amino (N)- and carboxyl (C)-termini of sequences are also shown. The fusion loop at aa. 820 to 830 is shown in yellow.

As shown in Figure 1A, six N-X-S or N-X-T sequons are encoded by the RVFV M-segment, where N is asparagine, X is any amino acid, S is serine, and T is threonine: *i.e.*, N88 (78-kD), N438 (78-kD and Gn), N794 (Gc), N829 (Gc), N1035 (Gc), and N1077 (Gc). The sites N88 and N438 are *N*-glycosylated in the 78-kD protein, and N438 is *N*-glycosylated in the Gn protein [13,14]; however, NSm is not *N*-glycosylated [14]. A previous study indicated that the Gc protein is *N*-glycosylated at three sites [13]. The recently solved crystal structure of Gc revealed architectural similarity with the envelope proteins of flaviviruses and alphaviruses, and it is categorized as a class II fusion protein. Similar to flaviviruses and alphaviruses [15], RVFV Gc consists of three domains: domain I (aa. 691–759, 852–901 and 981–1024), domain II (759–852, 901–981), and domain III (1024–1120) [16]. The fusion loop is located within domain II (aa. 820–830), which includes the N829 sequon. In the crystal, Gc forms head-to-tail homodimers, in which the fusion loop is buried by the domain III. An assembly model of Gn and Gc indicated that domain II, along with the fusion loop, may potentially be covered by the Gn surface protein [17].

The C-type lectin, dendritic cell specific intercellular adhesion molecule 3 (ICAM-3) grabbing non-integrin (DC-SIGN), was identified as a receptor for RVFV [18]. On the other hand, liver/lymph node-specific ICAM-3-grabbing non-integrin (L-SIGN or DC-SIGNR), a homologue of DC-SIGN, does not support the infectivity of RVFV or La Crosse virus (genus *Orthobunyavirus*) [19]. L-SIGN and DC-SIGN share 77% of their amino acid identity, but they are expressed on different cell types [20]. DC-SIGN is expressed on dendritic cells and some macrophages, whereas L-SIGN is expressed on the endothelial cells of liver and lymph node sinuses, as well as the endothelial cells lining capillaries of the placenta [20]. Because the *N*-glycans on the envelope glycoproteins (Gn and Gc) are ligands for the C-type lectin, they are expected to play an important role in RVFV infection via DC-SIGN.

The usage of each Gc sequon for *N*-glycosylation has not been determined. Furthermore, the requirement of RVFV Gn/Gc *N*-glycosylation for viral infection via DC-SIGN is not known. Using mutagenesis and reverse genetics, we aimed to identify the *N*-glycosylation sites utilized by RVFV Gc among the four potential sites (N794, N829, N1035, and N1077), and determine their individual role in viral infection via DC-SIGN.

## 2. Materials and Methods

### 2.1. Media, Cells, and Viruses

Minimum Essential Medium (MEM)-alpha supplemented with 10% fetal bovine serum (FBS) (Life Technologies, Carlsbad, CA, USA), penicillin (100 U/mL), streptomycin (100 µg/mL), and hygromycin B (600 µg/mL) was used to maintain BHK/T7-9 cells that express the T7 RNA polymerase [21]. Vero E6 cells (ATCC C1008) were grown in Dulbecco's Modified Eagle Medium (DMEM) supplemented with 10% FBS, penicillin (100 U/mL), and streptomycin (100 µg/mL). A Jurkat parental cell line and Jurkat cells stably expressing human DC-SIGN (Jurkat-DC-SIGN) or L-SIGN (Jurkat-L-SIGN) were kindly provided by Dr. Rafael Delgado (Molecular Microbiology Laboratory, Hospital Universitario 12 de Octubre, Madrid, Spain) [22]. Roswell Park Memorial Institute (RPMI) media supplemented with 10% FBS, penicillin (100 U/mL), and streptomycin (100 µg/mL) was used to grow the Jurkat cells, Jurkat-DC-SIGN, and Jurkat-L-SIGN cells. The RVFV vaccine strain MP-12 was derived from two passages of MP-12 lot 7 vaccine [23] in Vero cells. Recombinant RVFV MP-12 (rMP-12) and the corresponding *N*-glycosylation mutants (N438Q, N794Q, N1035Q, and N1077Q) were recovered by reverse genetics [24]. Plaque assay using Vero E6 cells was performed to determine viral titers [25].

### 2.2. Plasmids

To generate rMP-12 mutants encoding either N438Q, N794Q, N1035Q, N1077Q, N438Q/N729Q, N438Q/N1035Q or N438Q/N1077Q, we constructed seven plasmids: pProT7-vM(+)N438Q, pProT7-vM(+)N794Q, pProT7-vM(+)N1035Q, pProT7-vM(+)N1077Q, pProT7-vM(+)N438Q/N794Q, pProT7-vM(+)N438Q/N1035Q or pProT7-vM(+)N438Q/N1077Q by site-directed mutagenesis using PfuUltra High-Fidelity DNA polymerase (Agilent Technologies, La Jolla, CA, USA) according to the manufacturer’s instructions. The plasmids encode a point non-synonymous mutation (N to Q) at the indicated asparagine position (aa.1 represents the first methionine of M-segment open reading frame). The presence of individual mutations was confirmed by sequencing. To analyze the *N*-glycosylation of Gc, we modified pCAGGS-vG to encode all of N438Q, N729Q, N829Q, N1035Q, and N1077Q mutations (pCAGGS-vG-Gly-null). Then, one of those sequons was encoded in the following plasmids: pCAGGS-vG-N438(+), pCAGGS-vG-N794(+), pCAGGS-vG-N829(+), pCAGGS-vG-N1035(+), and pCAGGS-vG-N1077(+).

### 2.3. Precipitation of Gn/Gc by Concanavalin A Beads

Human embryonic kidney 293 cells (5 × 10^6^ cells) were transfected with pCAGGS-vG or the mutants (2 µg) by TransIT-293 (Mirus Bio LLC., Madison, WI, USA) according to the manufacturer’s instructions. At 48 h post transfection, cells were washed once with PBS, and harvested in RIPA buffer (150 mM NaCl, 50 mM Tris-HCl, 1% NP-40, 0.5% sodium deoxycholate, 0.1% sodium dodecyl sulfate) containing 1 mM of CaCl_2_, MgCl_2_, and MnCl_2_. Samples were mixed with 20 µL of agarose bound concanavalin A (AL-1003, Vector Laboratories, Burlingame, CA, USA), and further incubated at 4 °C for 16 h. After washing 3 times with phosphate buffered saline (PBS) with CaCl_2_ and MgCl_2_, each sample was re-suspended in denaturing buffer, and heated at 95 °C for 5 min. Samples were either not treated, or treated with 1000 units of PNGase F or Endo H (New England BioLabs, Ipswich, MA, USA) for 24 h at 37 °C. Then, samples were boiled in 2× sodium dodecyl sulfate (SDS) sample buffer and separated in 12% SDS-polyacrylamide gel electrophoresis (PAGE).

### 2.4. Western Blotting

Samples were analyzed by SDS-PAGE under reducing conditions. Western blot analysis was performed, as described previously [26]. Anti-RVFV Gn mouse monoclonal antibody (4D4) or anti-RVFV Gc rabbit polyclonal antibody (CAT#4521, ProSci, Inc., Poway, CA, USA) were used for the detection of RVFV Gn or Gc, respectively.

### 2.5. Radiolabeling of Virus Particles

Vero E6 cells were infected with rMP-12 or the mutants at a multiplicity of infection (MOI) of 0.1 to 3 at 37 °C. Cells were incubated for 30 min at 37 °C with MEM lacking methionine/cysteine and l-glutamine, which was complemented with 1% dialyzed FBS, 20 mM l-glutamine, penicillin (100 U/mL) and streptomycin (100 µg/mL) before the [^35^S] labeling of polypeptides. Trans [^35^S] label metabolic reagent (MP Biomedicals, Santa Ana, CA, USA) was added to infected cells at 1 hour post infection (hpi) (single asparagine mutant experiment) or 4 hpi (double asparagine mutant experiment) to radiolabel virus particles. At 16 hpi, supernatant was harvested and clarified by low-speed centrifugation [4800 revolutions per minute (rpm) for 5 min]. Virus particles were then immunoprecipitated using anti-RVFV antibody, and washed four times in PBS. Samples were then re-suspended in 2× sample buffer containing 5% mercaptoethanol, and boiled for 10 min at 100 °C. Then, Gn and Gc mobility was analyzed by SDS-PAGE and subsequent autoradiography.

### 2.6. Infectivity of rMP-12 or the N-Glycan Mutants in Jurkat-DC-SIGN or Jurkat-L-SIGN Cells

Jurkat, or Jurkat-DC-SIGN cells (1 × 10^6^ cells) were mock-infected or infected with 6.3 × 10^6^ RNA copies of rMP-12 or the mutants (MOI = 3.6). After infection, cells were incubated at 37 °C for 6 h. Cells were then fixed with 4% paraformaldehyde for 30 min at 4 °C, followed by washing with PBS. Then, cells were permeabilized with permeabilization buffer (Affimetrix eBioScience, San Diego, CA, USA) at 4 °C for 25 min. Then, cells were incubated with anti-RVFV mouse ascite and Alexa Fluor 488-conjugated anti-green fluorescent protein (GFP) rabbit antibody (Life Technologies, Carlsbad, CA, USA) diluted in permeabilization buffer at 4 °C for 40 min. As an antibody control for GFP detection, Alexa Fluor 488-conjugated normal rabbit immunoglobulin (Ig)G (EMD Millipore, Billerica, MA, USA) was used. Cells were washed twice with permeabilization buffer, and the cells were incubated at 4 °C for 40 min with Alexa Fluor 647-conjugated goat anti-mouse IgG (Life Technologies). Permeabilization buffer was used to wash cells three times, and then cells were resuspended in fluorescence-activated cell sorting (FACS) buffer. Cells were analyzed by flow cytometry on the Canto or LSRII Fortessa (BD Biosciences, San Jose, CA, USA) in the UTMB Flow Cytometry and Cell Sorting Core Facility using FACSDiva software (version 8.0.1, BD Biosciences) and analyzed in FlowJo version 9.7 (TreeStar, Ashland, OR, USA).

### 2.7. Statistical Analysis

The statistical analyses were performed using the GraphPad Prism version 6.05 for Windows (GraphPad Software Inc., La Jolla, CA, USA). The unpaired Student’s *t* test was used for the comparison of two groups.

### 2.8. Ethics Statement

All the recombinant DNA and RVFV were created upon the approval of the Notification of Use by the Institutional Biosafety Committee at UTMB.

## 3. Results

### 3.1. RVFV Gc N829 Is N-P-S Sequon and Is Located at Fusion Loop

As shown in Figure 1A, RVFV encodes six distinct N-X-S or N-X-T sequons in the M-segment: N88 (78-kD: N-I-T), N438 (78-kD/Gn: N-G-S), N794 (Gc: N-E-T), N829 (Gc: N-P-S), N1035 (Gc: N-L-T), and N1077 (Gc: N-G-T). The proline (P) at the X-site does not grant access of the oligosaccharyltransferase (OST) to the asparagine, and thus, N-P-S/T sequons cannot be *N*-glycosylated [27,28,29]. The N-P-S sequon at RVFV Gc N829 is located in the fusion loop, and an alignment with closely related phleboviruses revealed the N-P-S sequon was also found in TOSV and PTV (Figure 1B). On the other hand, the RVFV Gc N1035 could be aligned with SFSV (N1180), TOSV (N1180), and PTV (N1161), while the sequon corresponding to RVFV N794 was present in SFSV, and TOSV Gc, but not in PTV Gc. The sequon corresponding to N1077 was found in neither SFSV, TOSV, nor PTV. These results indicate the evolutional conservation of N794 and N1035 among RVFV, Sicilian, Naples serocomplexes, and the N1077 sequon was uniquely found in RVFV.

### 3.2. RVFV Gc N794, N1035, and N1077, but Not N829, Are N-Glycosylated

**Figure 2:**
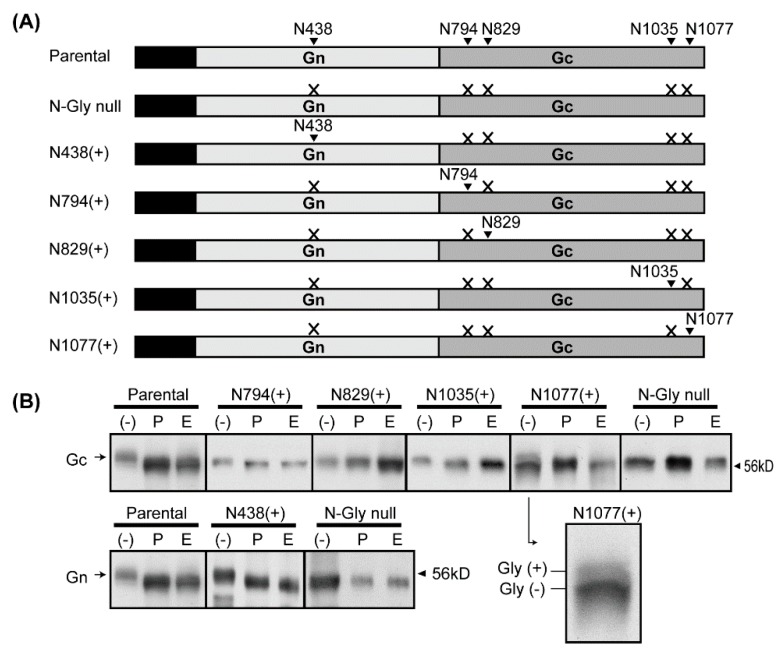
Mapping of *N*-glycosylation sites for RVFV Gc. (**A**) Schematics of the Medium (M)-segment open reading frame (ORF) encoded in the following plasmids: Parental (pCAGGS-vG) , N-Gly null (pCAGGS-vG-Gly-null), N438(+) (pCAGGS-vG-N438(+)), N794(+) (pCAGGS-vG-N794(+)), N829(+) (pCAGGS-vG-N829(+)), N1035(+) (pCAGGS-vG-N1035(+)), or N1077(+) (pCAGGS-vG-N1077(+)). N-to-Q mutations were made in the X-sites, and the remaining single asparagine site is shown with an arrowhead; (**B**) 293 cells were transfected with plasmids shown in A. Then, Gn/Gc were precipitated with concanavalin agarose beads, and subsequently detected by anti-Gc antibody (top panels) or anti-Gn antibody (bottom panels). Samples were either not treated (-), or treated with Peptide-N-Glycosidase F (PNGase F) (P) or Endoglycosidase H (E). An image of untreated N1077(+) lane is enlarged to show both glycosylated (Gly(+)) and unglycosylated (Gly(−)) bands.

To determine the *N*-glycosylation status of each sequon encoded by RVFV Gc, we generated plasmids encoding RVFV M-segment open reading frame (ORF) lacking all sequons (pCAGGS-vG-Gly-null), or encoding only one sequon (Figure 2A). Human embryonic kidney 293 cells were transfected with parental pCAGGS-vG, or the mutant: pCAGGS-vG-Gly-null, pCAGGS-vG-N438(+), pCAGGS-vG-N794(+), pCAGGS-vG-N829(+), pCAGGS-vG-N1035(+), or pCAGGS-vG-N1077(+). At 48 h post transfection, cell lysates were harvested, and incubated with concanavalin A agarose beads. After washing, precipitates were subjected to Western blot analysis using anti-Gn or anti-Gc antibody (Figure 2B). *N*-glycans are classified into three types, based on the sugar structure attached to the common core sugar structure of three mannoses (Man) and two *N*-acetyl-glucosamines (GlucNAc) linked to the asparagine residue: (a) high mannose: only mannoses are attached to the core; (b) hybrid: the Manα1-6 arm is attached to only mannoses, and the Manα1-3 arm is attached to “antennae” (GlucNAc); and (c) complex: two “antennae” (GlucNAc) are attached via the Manα1-3 and Manα1-6 arms. To evaluate the presence of *N*-glycosylation, paired precipitated samples were treated with either PNGase F (cleaves between the innermost GlcNAc and the asparagine of high mannose-type, hybrid-type, and complex-type *N*-glycans) or Endo H (cleaves between two GlucNAc in the core of high mannose-type and some hybrid-type *N*-glycans) [30]. Parental Gn and Gc showed increased migration after PNGase F or Endo H enzyme treatments, confirming that RVFV Gn and Gc are *N*-glycosylated. As expected, the Gn and Gc lacking all sequons (*N*-Gly null) did not show migration changes after either enzyme treatment. N438(+) Gn reacted to both PNGase F and Endo H treatment, and the bands showed increased migration. N794(+) and N1035(+) Gc also reacted to both PNGase F and Endo H treatments, and bands showed increased migration compared to non-treated samples. N829(+) showed a similar migration pattern to *N*-Gly null, and no change in band migration was observed after either enzyme treatment. Interestingly, N1077(+) showed doublet bands, and the slower migrating band was not present after either PNGase F or Endo H treatment. Our results demonstrated that Gn N438, and Gc N794, N1035, and N1077 are *N*-glycosylated, and all are high mannose-type (or hybrid-type) *N*-glycans.

### 3.3. Generation of Recombinant RVFV Encoding N-to-Q Substitutions at One or Two N-Glycan Sequons

**Figure 3:**
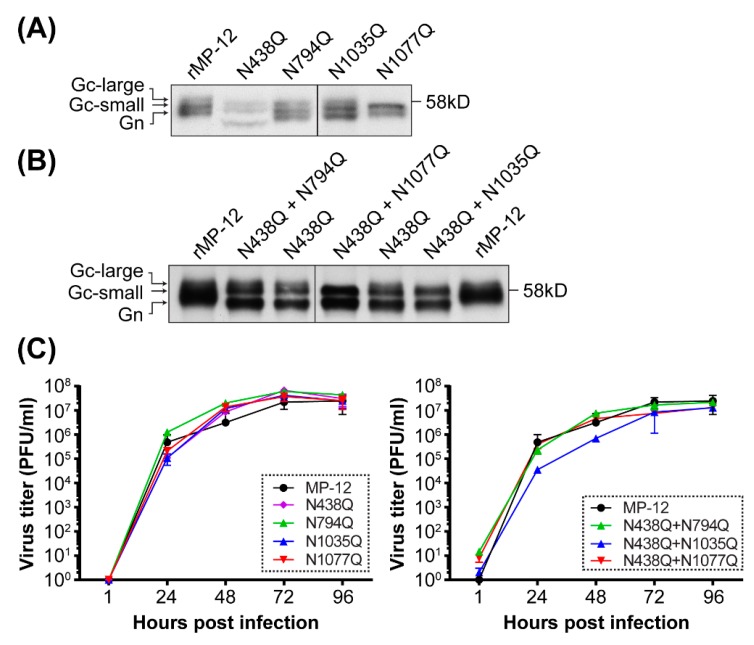
Generation of recombinant MP-12 encoding asparagine (N)-to-glutamine (Q) mutation(s) in Gn/Gc sequons. The Gn/Gc migration patterns of rMP-12, or that encoding N-to-Q mutation either in Gn or Gc (N438Q, N794Q, N1035Q, and N1077Q mutants) (**A**) or that encoding N-to-Q mutations both in Gn and Gc (N438Q/N794Q, N438Q/N1035Q, and N438Q/N1077Q mutants) (**B**). Vero E6 cells were infected with rMP-12 or the mutants at a multiplicity of infection (MOI) of 0.1 to 3, and metabolically labeled with [^35^S] methionine/cysteine from 1 to 16 hours post infection (hpi). The cleared culture supernatants were subjected to immunoprecipitation using anti-Rift Valley fever virus (RVFV) antibody. Precipitated virions were analyzed by 7.5% sodium dodecyl sulfate- polyacrylamide gel electrophoresis (SDS-PAGE) and autoradiography. Gc-large: slow migrating Gc; Gc-small: fast migrating Gc; (**C**) Virus growth kinetics in Vero cells. Vero cells were infected with indicated rMP-12 mutants at an MOI of 0.01. Virus titers were determined at 1, 24, 48, 72 and 96 hpi. Means +/− standard deviations of three independent experiments are shown.

To understand the role of the individual *N*-glycans of RVFV Gn/Gc, we replaced the asparagine (N) of each sequon with a glutamine (Q), and generated recombinant RVFV MP-12 strains, encoding N794Q, N1035Q, N1077Q, N438Q/N794Q, N438Q/N1035Q, or N438Q/N1077Q (Figure 3A). The N-to-Q substitution was used to minimize any structural changes due to mutagenesis because glutamine, H2N-CO-(CH2)2-CH(NH2)-COOH, is a polar and neutral amino acid, which is similar to asparagine, H2N-CO-(CH2)-CH(NH2)-COOH. Next, we analyzed migrations of Gn and Gc bands using immunoprecipitated radiolabeled virions. As expected, all rMP-12 mutants encoding N438Q (e.g., N438Q, N438Q/N794Q, N438Q/N1035Q, and N438Q/N1077Q) showed a fast migrating Gn band (Figure 3A,B). Gc displayed as doublet bands (Gc-large and Gc-small), while Gc-large was not present in N1077Q, N438Q/N1035Q, or N438Q/N1077Q. Since heterogeneous N-glycosylation occurs at N1077 (Figure 2B), Gc-large for N1035Q could be *N*-glycosylated at N794 and N1077, and Gc-large for N794Q could be *N*-glycosylated at N1035 and N1077. Though the N438Q/N1035Q mutant could be also *N*-glycosylated at N794 and N1077, we could not detect a distinct Gc-large band (Figure 1B). All single or double mutants replicated efficiently in Vero cells and showed similar growth kinetics to parental rMP-12 (MOI = 0.01) (Figure 3C). We also tried to rescue rMP-12 encoding N1035Q/N1077Q, but no viable viruses could be recovered after three independent attempts. The rMP-12 encoding N829Q was viable and replicated efficiently in Vero cells (data not shown), but was not further studied because N829 was not utilized for *N*-glycosylation (Figure 2B).

### 3.4. RVFV Gn and Gc N-Glycans Redundantly Support Viral Infection via DC-SIGN

**Figure 4:**
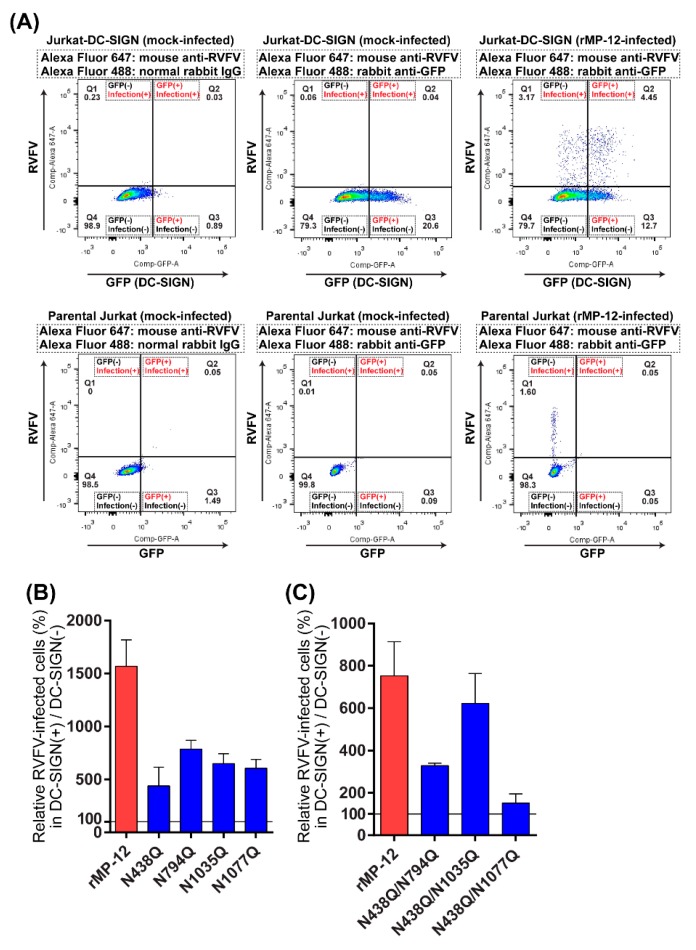
Infectivity of recombinant MP-12 encoding a glutamine (Q) in place of an asparagine (N) at N-X-S/T sequon(s) in Gn or Gc. (**A**) Jurkat-DC-SIGN cells stably co-expressing green fluorescent protein (GFP) and human DC-SIGN (~17% in cell population) (top panels), or parental Jurkat cells, which express neither DC-SIGN nor GFP (bottom panels), were mock-infected (left and center panels) or infected with rMP-12 (right panels) at a multiplicity of infection (MOI) of 3.6. At 6 hpi, cells were fixed, permeabilized, and then stained with a cocktail of mouse anti-RVFV antibody and Alexa Fluor 488-conjugated rabbit anti-GFP antibody, or a cocktail of mouse anti-RVFV antibody and Alexa Fluor 488-conjugated normal rabbit IgG. Subsequently, cells were stained with Alexa Fluor 647-conjugated goat anti-mouse IgG, and analyzed by flow cytometry. Since permeabilized Jurkat-DC-SIGN cells showed poor intrinsic GFP signals, as shown with Alexa Fluor 488-conjugated normal rabbit IgG (left top panel), rabbit anti-GFP antibody (center and right top panels) was used to detect GFP signals of permeabilized Jurkat-DC-SIGN cells. Q1, GFP-negative (DC-SIGN-negative) and RVFV-infected cell population; Q2, GFP-positive (DC-SIGN-positive) and RVFV-infected cell population; Q3, GFP-positive (DC-SIGN-positive) and uninfected cell population; Q4, GFP-negative (DC-SIGN-negative) and uninfected cell population; (**B**,**C**) Jurkat-DC-SIGN cells were infected with rMP-12 or that lacking one (**B**) or two sequons (**C**). Relative number of RVFV-infected cells in the GFP-positive cell population (Q2/(Q2+Q3)) normalized to that of RVFV-infected cells in the GFP-negative cell population (Q1/(Q1+Q4)) are shown. Graphs represent mean + standard deviations for three independent experiments.

Next, we analyzed the function of Gn and Gc *N*-glycans during infection of host target cells. A previous study indicated that a C-type lectin, DC-SIGN (CD209), which is expressed on dendritic cells and macrophages, functions as a receptor for phleboviruses [18]. Jurkat cells stably expressing human DC-SIGN [22] that also co-express GFP in up to 17% of the population (Figures S1 and S2) were used to determine infectivity via the lectin [22]. Since GFP and DC-SIGN were overall co-expressed, we analyzed cell populations expressing GFP to analyze RVFV infection via DC-SIGN. Though Jurkat-DC-SIGN cells expressed intrinsic GFP signals, the signal was very weak in the flow cytometry data analysis (Figure 4A, left top panel). Therefore, anti-GFP antibody was used to detect a population expressing GFP (Figure 4A, center and right top panels). To determine the relative infection efficiency of rMP-12, Jurkat-DC-SIGN cells were either mock-infected or infected with rMP-12 at an MOI of 3.6 (measured in Vero E6 cells). At 6 hpi, cells were fixed and permeabilized, and we analyzed the infectivity of virus in GFP-positive and in GFP-negative cells.

Both Jurkat-DC-SIGN and parental Jurkat cells could be infected with rMP-12 (Figure 4A, right top and right bottom panels). Thus, it was important to analyze viral infection via DC-SIGN, over the nonspecific viral infection in Jurkat cells. To analyze viral infection via DC-SIGN, relative number of RVFV-infected cells in the GFP-positive cell population (Q2/(Q2+Q3)) was normalized to that of RVFV-infected cells in the GFP-negative cell population (Q1/(Q1+Q4)) (background level infectivity in Jurkat cells: 100%). This value most probably represents the infectivity of rMP-12 via DC-SIGN, over the nonspecific infectivity independent of DC-SIGN. Using this approach, we measured the infectivity of rMP-12 lacking one or two sequons, as shown in Figure 4B,C.

The rMP-12-positive cells increased more in DC-SIGN-positive cells (7.5 to 15-fold) compared to cells not expressing DC-SIGN (Figure 4B,C). All single asparagine mutants (N438Q, N794Q, N1035Q, or N1077Q) still showed an increased infection in DC-SIGN-positive cells (N438Q: 4.4-fold, N794Q: 7.9-fold, N1035Q: 6.5-fold, and N1077Q: 6.1-fold). Double asparagine mutants, N438Q/N794Q (3.3-fold) and N438Q/N1035Q (6.2-fold) still had an increased infection in DC-SIGN-positive cells, while N438Q/N1077Q (1.5-fold) no longer showed enhanced infectivity in DC-SIGN-positive cells. Taken together, it is indicated that either Gn N438, Gc N794 + N1077, or Gn N1035 + N1077 can increase viral infection via DC-SIGN. Thus, Gn and Gc N-glycans redundantly support virus infection via DC-SIGN.

## 4. Discussion

Modification of proteins by *N*-glycosylation occurs co-translationally in the rough endoplasmic reticulum (ER) by the en bloc transfer of an oligosaccharide *N*-glycan precursor onto the N-X-S/T sequons in the nascent polypeptide by the OST complex. Subsequently, the *N*-glycans are processed (e.g., trimming or addition of sugar residues) in the ER and the Golgi by glycosidases and glycosyltransferases. In this study, we determined that three (N794, N1035, and N1077) out of four Gc sequons are *N*-glycosylated. The presence of proline at the X-site (N-X-S/T) of the sequons inhibits *N*-glycosylation [27,28,29], and, the N-P-S sequon at RVFV Gc (N829) was not *N*-glycosylated. Our results also indicated that RVFV Gn and Gc *N*-glycans are high mannose or hybrid-type *N*-glycans, susceptible to Endo H treatment. *N*-glycosylation is catalyzed by either the STT3A or STT3B subunit of the OST complex of mammalian cells [31]. In general, *N*-glycosylation of nascent polypeptides occurs co-translationally by the STT3A-OST-complex, while posttranslational *N*-glycosylation is less common [32]. STT3B-mediated posttranslational glycosylation occurs near the C-terminal of unfolded proteins when the sequon is skipped by the STT3A-OST complex [31]. We observed that *N*-glycosylation of RVFV Gc at N1077 occurs only partially, which leads to the generation of Gc-large and Gc-small. Heterogeneous *N*-glycosylation of Gc suggests that N-glycosylation at N1077, which is located at 121 amino acids upstream of the C-terminus, occurs post-translationally.

We analyzed viral infection via DC-SIGN, using Jurkat-DC-SIGN cells. The Jurkat-DC-SIGN cells expressed GFP in up to 17% of cell population, and GFP-positive cells were overall co-expressed with DC-SIGN (Figure S2). In the samples prepared at an early stage of infection (6 hpi), we compared the number of infected cells in GFP-positive population, and GFP-negative population. MP-12 infection occurred preferably in GFP-positive population, indicating that DC-SIGN acts as a receptor for RVFV [18]. The N438Q/N1077Q mutant no longer retained enhanced infectivity via DC-SIGN, while the N438Q, N794Q, N1035Q, N1077Q, N438Q/N794Q, and N438Q/N1035Q mutants showed an increased viral infection via DC-SIGN. Thus, our results indicated that Gn N438 or Gc N1077 play an important role in viral infection via DC-SIGN, and that Gn and Gc N-glycans redundantly support virus infection via DC-SIGN.

It should be noted that recombinant RVFV lacking one or more sequons may have unpredictable effects on the usage of other sequons. We observed reduced infectivity of the N438Q or N1035Q mutants in Jurkat-DC-SIGN cells, while the N438Q/N1035Q double mutation had little impact on viral infection in those cells. In addition, the N438Q/N1035Q mutant did not show a distinct Gc-large band, unlike the N438Q/N794Q mutant. Since the Gc-large for the N438Q/N1035Q mutant could be *N*-glycosylated at N794 and N1077, the *N*-glycosylation status either at N794 or N1077 may be altered in the N438Q/N1035Q mutant. Effects of N-terminal *N*-glycosylation on C-terminal sites have been studied in the rabies virus glycoprotein (GP) [33]. The rabies virus GP is type I membrane glycoprotein and encodes three sequons: N37, N247, and N319. The GP lacking N37 and N319 was largely susceptible to Endo H, while intact GP or GP lacking only N37 were resistant to Endo H. Thus, the presence of an *N*-glycan at one site can affect the processing of an *N*-glycan at another site. Another study showed that the insertion of a new sequon (N58) in human plasminogen activator influences the processing of the *N*-glycosylation at N117 [34]. While this is beyond the scope of the current study, determination of carbohydrate chains at each asparagine residue will elucidate this mechanism of *N*-glycosylation modification during Gn/Gc maturation of each mutant.

We also analyzed the infectivity of MP-12 and the mutants in Jurkat cells expressing L-SIGN (Jurkat-L-SIGN cells) (Figure S3) [22]. However, we observed only a 1.2-fold augmentation of MP-12 infection via L-SIGN, in contrast to the 7.5–15-fold augmentation of infectivity via DC-SIGN. This result is consistent with the previous study by Hofmann *et al.* that demonstrated mosquito-borne RVFV or La Crosse virus (genus *Orthobunyavirus*) specifically utilize DC-SIGN, but not L-SIGN, while tick-borne severe fever with thrombocytopenia syndrome virus (SFTSV; genus *Phlebovirus*) uses both DC-SIGN and L-SIGN for entry [19]. Both DC-SIGN and L-SIGN are homotetrameric type II membrane proteins and retain 77% amino acid identity [20]. L-SIGN selectively binds to the trisaccharide Manα1-3(Manα1-6)Manα1 on high mannose glycans, while DC-SIGN binds to high mannose glycans (preferably with eight or nine mannoses) or fucose-containing structures including the Lewis-X trisaccharide: *i.e.*, Galβ1-4(Fucα1-3)GlcNAc [35,36,37]. Though both DC-SIGN and L-SIGN bind to high mannose-type *N*-glycans, the pH-dependent release of the oligosaccharide ligand by L-SIGN is not as efficient as DC-SIGN [37], which might explain the poor infectivity of RVFV via L-SIGN.

Though the infectivity was not high, parental Jurkat cells, which do not express those C-type lectins, could be also infected with MP-12. It was shown that RVFV entry is inhibited in Chinese hamster ovary (CHO) cells pgs-745 mutant (deficient in glycosaminoglycan synthesis) and the pgsD-677 mutant (deficient in synthesis of heparin sulfate: HS), or in CHO cells pretreated with heparinases [38]. Thus, HS also plays a role in RVFV entry. Since Jurkat cells synthesize HS [39], MP-12 infection of parental Jurkat cells is most likely mediated by HS. Indeed, in another study where DC-SIGN was expressed in Raji cells, a B-cell lymphoma cell line deficient in HS synthesis [40], RVFV infection was supported [18], indicating that RVFV entry via DC-SIGN does not require HS. In our study, an increased MP-12 infection occurred in Jurkat-DC-SIGN cells in the presence of both DC-SIGN and HS. However, further study is required to understand if the co-expression of DC-SIGN and HS synergically facilitates the entry of RVFV.

We also noted that RVFV Gn/Gc lacking all *N*-glycans could be still expressed without showing unstable characteristics. The N-to-Q mutation of Bunyamwera virus (genus *Orthobunyavirus*) Gn N60 resulted in the loss of immunoreactivity with an anti-Gc monoclonal antibody [41]. Further, the N-to-Q mutation of Hantaan virus (genus *Hantavirus*) Gn N134 resulted in poor accumulation of Gn and poor immunoreactivity to anti-Gc monoclonal antibodies [42]. Thus, RVFV *N*-glycans might be dispensable for protein stability. On the other hand, rMP-12 encoding N1035Q/N1077Q, N438Q/N794Q/N829Q/N1035Q/N1077Q, or N794Q/N829Q/N1035Q/N1077Q were not rescued successfully. Thus, *N*-glycans may play a role in combination to form a functional Gn/Gc complex for viral assembly.

In addition to Gn and Gc, RVFV also encodes 78 kD proteins, which are incorporated into virions matured from mosquito cells, but not those from mammalian cells [12]. Though the 78 kD protein shares the amino acid sequence with Gn, including the N438 sequon, it makes a distinct structure from the Gn and does not function as a precursor for Gn production [43,44]. The N-terminus encodes the N88 sequon, which is unique to 78 kD protein. A lack of 78 kD affects viral dissemination in mosquitoes [11,45,46], and it may have a distinct role from Gn and Gc in viral entry mechanism. Future studies involving the N-glycosylation of 78 kD and its potential role in viral entry will prove valuable in further elucidating the function of this protein.

## 5. Conclusions

We demonstrated the presence of *N*-glycans in Gn (N438) and Gc (N794, N1035, and N1077). RVFV Gc consists of two distinct *N*-glycoforms (Gc-large and Gc-small), due to heterogeneous *N*-glycosylation at N1077. We found that RVFV infection via DC-SIGN occurs in a redundant manner through Gn and Gc, and that *N*-glycans at Gn N438 and Gc N1077 play an important role in viral infection via DC-SIGN. Our study will support a better understanding of the post-translational *N*-glycan modification of Gn/Gc and its role in progeny infection.

## Supplementary Materials

The following are available online at www.mdpi.com/1999-4915/8/5/149/s1, Figure S1: Co-expression of GFP and DC-SIGN in Jurkat-DC-SIGN cells, Figure S2: Populations of cells co-expressing GFP and DC-SIGN in Jurkat-DC-SlGN cells, Figure S3: Infectivity of recombinant MP-12 encoding a glutamine (Q) in place of an asparagine (N) at N-X-S/T sequon(s) in Gn or GC in Jurkat-L-SIGN cells.

## Abbreviations

The following abbreviations are used in this manuscript:

BHK	Baby hamster kidney
DC-SIGN	Dendritic cell specific ICAM-3 grabbing non-integrin
EDTA	Ethylenediaminetetraacetic acid
FACS	Fluorescence-activating cell sorting
FBS	Fetal bovine serum
G	Glycine
GFP	Green fluorescent protein
HHS	U.S. Department of Health and Human Services
I	Isoleucine
L	Leucine
L-segment	Large-segment
L-SIGN	Liver/lymph node-specific ICAM-3-grabbing non-integrin
M-segment	Medium-segment
MOI	Multiplicity of infection
N	Asparagine
NIAID	National Institute of Allergy and Infectious Diseases
NIH	National Institutes of Health
OST	Oligosaccharyltransferase
P	Proline
PBS	Phosphate buffered saline
PTV	Punta Toro virus
Q	Glutamine
RIPA	Radioimmunoprecipitation assay buffer
RVF	Rift Valley fever
RVFV	Rift Valley fever virus
S	Serine
SDS-PAGE	Sodium dodecyl sulfate-polyacrylamide gel electrophoresis
SFSV	Sandfly fever Sicilian virus
SFTSV	Severe Fever with Thrombocytopenia Syndrome virus
S-segment	Small-segment
STT	Subunit of the oligosaccharyltransferase complex
T	Threonine
TOSV	Toscana virus
USDA	U.S. Department of Agriculture
