# Supplementary material for: N-Glycans on the Rift Valley Fever Virus Envelope Glycoproteins Gn and Gc Redundantly Support Viral Infection via DC-SIGN
Source: Viruses. 2016 May 23;8(5):149. doi: 10.3390/v8050149 (PMC4885104; doi:10.3390/v8050149)
Supplement: Supplementary file 1 [file viruses-08-00149-s001.pdf]

viruses

MDPI

Article

Supplementary material: N-Glycans on the Rift Valley Fever Virus Envelope Glycoproteins Gn and Gc Redundantly Support Viral Infection via DC-SIGN

Inaia Phoenix, Shoko Nishiyama, Nandadeva Lokugamage, Terence E. Hill, Matthew B. Huante Olga A. L. Slack, Victor H. Carpio, Alexander N. Freiberg and Tetsuro Ikegami

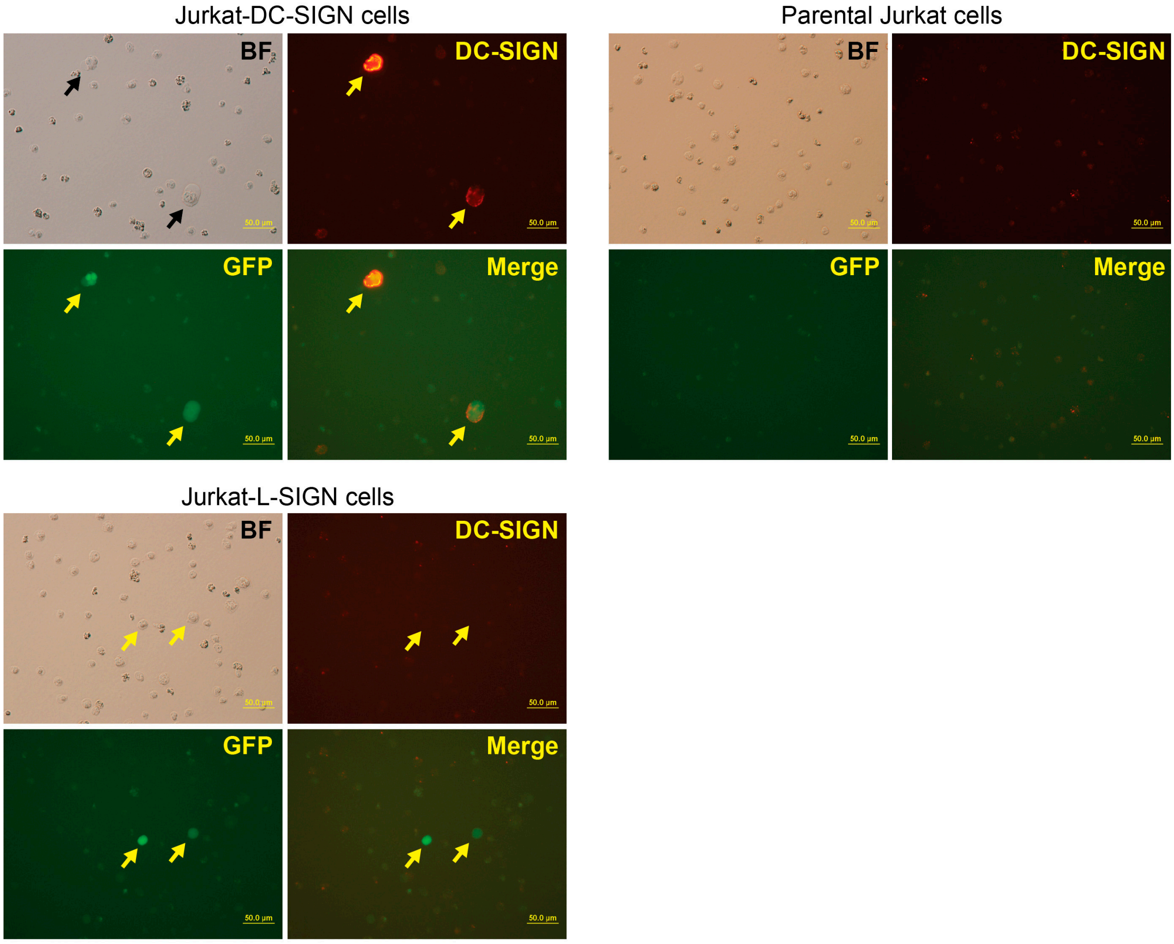

Figure Sl. Co-expression of GFP and DC-SIGN in Jurkat-DC-SIGN cells. Jurkat (parental), Jurkat-DC-SlGN, Jurkat-L-SlGN cells were fixed with 4% paraformaldehyde for 15 min on ice, and then, incubated with anti-human DC-SIGN rabbit monoclonal antibody (Cell Signaling Tech: #13193) for 16 hours at 40C. After washing with PBS, cells were stained with Alexa Fluor 594 goat anti-rabbit lgG (ThermoFisher: #A-11037) at 37 oc for 1 hour. Washed cells were observed in a chamber slide under Olympus IX71 fluorescense microscope. Jurkat-DC-SlGN and Jurkat-L-SlGN cells, but not parental Jurkat cells, express GFP intrinsically. BF (top left): bright field image, DC-SIGN (top right): Alexa Fluor 594 signals (red) specific to DC-SIGN, GFP (bottom left): cells expressing GFP intrinsically, Merge, (bottom right): merged image of DC-SIGN and GFP. Arrows show the location of GFP-positive cells.

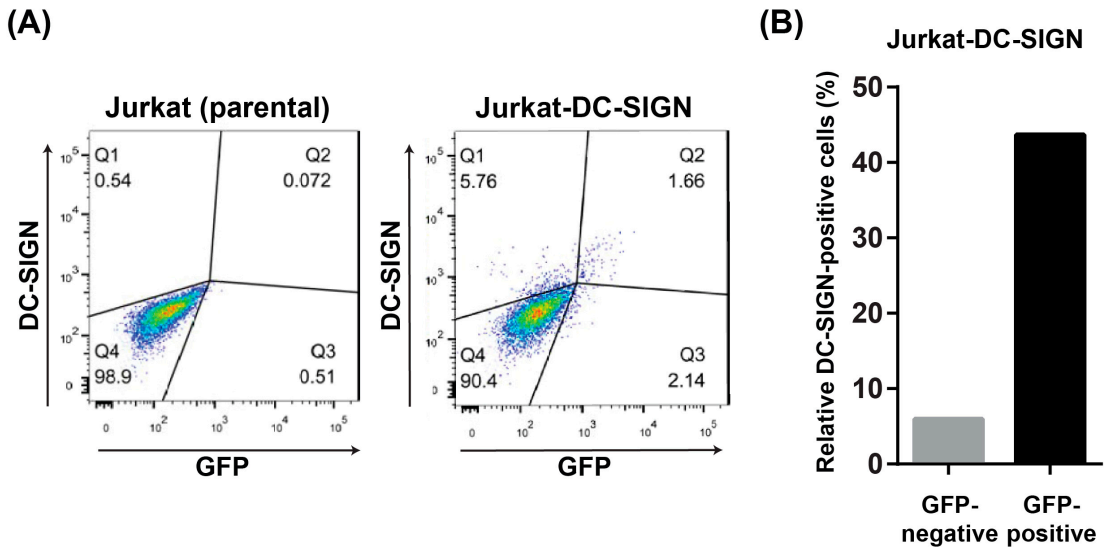

Figure S2. Populations of cells co-expressing GFP and DC-SIGN in Jurkat-DC-SlGN cells. Indirect staining of extracellular DC-SIGN using anti-human DC-SIGN rabbit monoclonal antibody (Cell Signaling Tech: #13193) followed by Alexa Fluor 647 goat anti-rabbit lgG (ThermoFisher: #A-121244) was performed on parental Jurkat cells and Jurkat-DC-SlGN cells. After extracellular staining, cells were fixed with 4% paraformaldehyde for 30 mins at 40C, and then permeabilized with permeabilization buffer (Affimetrix eBioScience) for 30 mins at 40C. After permeabilization, Alexa Fluor 488 anti-GFP mouse monoclonal antibody B-2 (Santa Cruz Biotechnology) was used to stain cells for 30 mins at 40C. Though Jurkat-DC-SlGN cells intrinsically express GFP, the GFP-positive population was not detected separately from GFP-negative cells in permeabilized samples, due to weak signals (Fig. Sl), without using anti-GFP antibody. Cells were washed three times with permeabilization buffer, resuspended in FACs buffer, and collected on a LSRII Fortessa (BD BioSciences) in the UTMB Flow Cytometry and Cell Sorting Core Facility using FACSDiva Software and analyzed in FlowJo version 9.7 (TreeStar). Fluorescence Minus One (FMO) controls for Alexa-647 and anti-GFP were done in parental Jurkat cells and Jurkat-DC-SlGN cells. (A) FACS images of parental Jurkat (left panel), and Jurkat-DC-SlGN cells (right panel). (B) Relative DC-SIGN-positive cells in GFP-negative cells or those in GFP-positive cells in Jurkat-DC-SlGN cells. It should be noted that GFP-positive cells (3.8%) and DC-SIGN-positive cells (7.42%) were detected in Jurkat-DC-SlGN cells, which was lower than those detected using Alexa Fluor 488-conjugated anti-GFP rabbit antibody (Fig. 4), probably due to decreased sensitivity of mouse anti-GFP antibody and anti-DC-SIGN antibodies in the co-staining protocol.

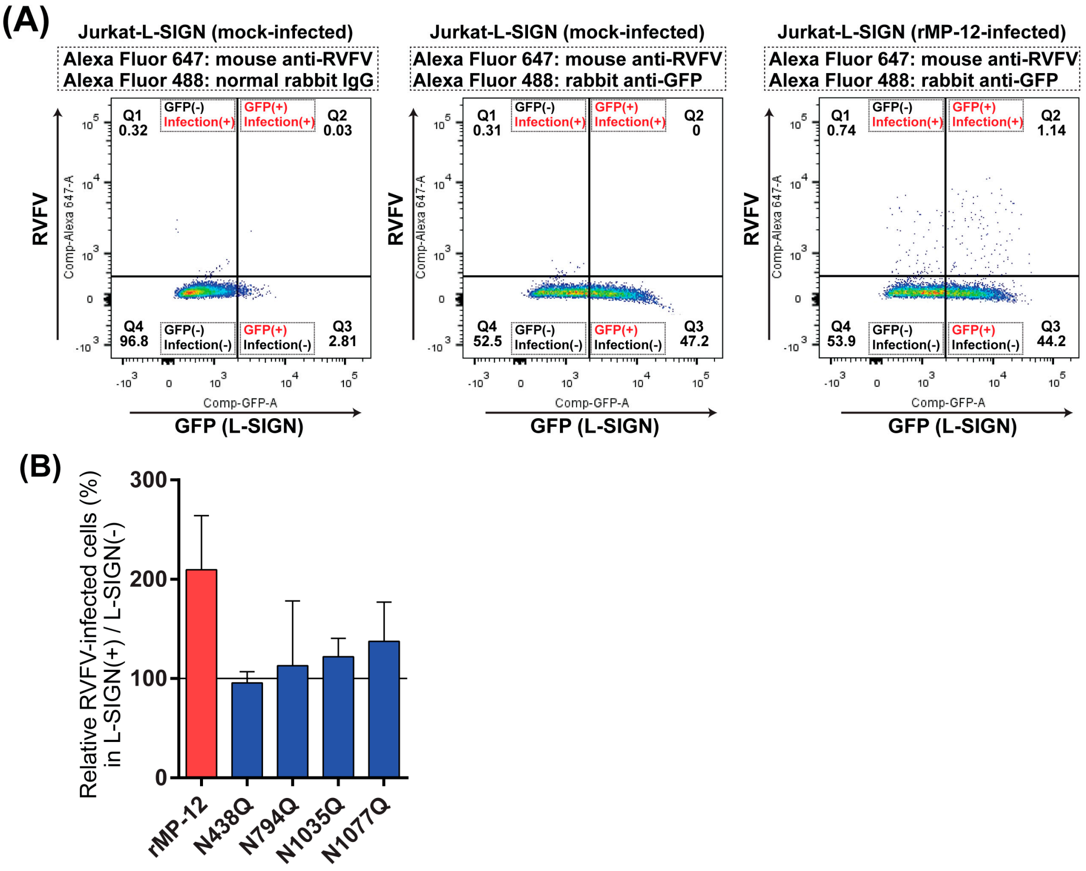

Figure S3. Infectivity of recombinant MP-12 encoding a glutamine (Q) in place of an asparagine (N) at N-X-S/T sequon(s) in Gn or GC in Jurkat-L-SIGN cells. (A) Jurkat-L-SIGN cells were mock-infected (left and center panels) or infected with rMP-12 at an MOI of 3.6 (right panel). At 6 hpi, cells were fixed, permeabilized, and then stained with a cocktail of mouse anti-RVFV antibody and Alexa Fluor 488-conjugated rabbit anti-GFP antibody (center and right panels), or a cocktail of mouse anti-RVFV antibody and Alexa Fluor 488-conjugated normal rabbit IgG (left panel). Then, cells were stained with Alexa Fluor 647-conjugated goat anti-mouse lgG, and analyzed by flow cytometry. Intrinsic GFP signal was weak, and thus, anti-GFP antibody was used to detect GFP-positive cells. QI, GFP-negative (L-SIGN-negative) and RVFV-infected cell population; Q2, GFP-positive (L-SIGN-positive) and RVFV-infected cell population; Q3, GFP-positive (L-SIGN-positive) and uninfected cell population; Q4, GFP-negative (L-SIGN-negative) and uninfected cell population. (B) Jurkat-L-SIGN cells were infected with rMP-12 or the mutants, as described. Relative number of RVFV-infected cells in the GFP-positive cell population normalized to that of RVFV-infected cells in the GFP-negative cell population LQI/(QI +Q4)J are shown. Graphs represent mean + standard deviations for 3 independent experiments.

© 2016 by the authors. Submitted for possible open access publication under the terms and conditions of the Creative Commons Attribution (CC-BY) license (http://creativecommons.org/licenses/by/4.0/).
